# Rank Diversity of Languages: Generic Behavior in Computational Linguistics

**DOI:** 10.1371/journal.pone.0121898

**Published:** 2015-04-07

**Authors:** Germinal Cocho, Jorge Flores, Carlos Gershenson, Carlos Pineda, Sergio Sánchez

**Affiliations:** 1 Instituto de Física, Universidad Nacional Autónoma de México, Mexico City, Mexico; 2 Centro de Ciencias de la Complejidad, Universidad Nacional Autónoma de México, Mexico City, Mexico; 3 Instituto de Investigaciones en Matemáticas Aplicadas y en Sistemas, Universidad Nacional Autónoma de México, Mexico City, Mexico; 4 Facultad de Ciencias, Universidad Nacional Autónoma de México, Mexico City, Mexico; Max Planck Institute for the Physics of Complex Systems, GERMANY

## Abstract

Statistical studies of languages have focused on the rank-frequency distribution of words. Instead, we introduce here a measure of how word ranks change in time and call this distribution *rank diversity*. We calculate this diversity for books published in six European languages since 1800, and find that it follows a universal lognormal distribution. Based on the mean and standard deviation associated with the lognormal distribution, we define three different word regimes of languages: “heads” consist of words which almost do not change their rank in time, “bodies” are words of general use, while “tails” are comprised by context-specific words and vary their rank considerably in time. The heads and bodies reflect the size of language cores identified by linguists for basic communication. We propose a Gaussian random walk model which reproduces the rank variation of words in time and thus the diversity. Rank diversity of words can be understood as the result of random variations in rank, where the size of the variation depends on the rank itself. We find that the core size is similar for all languages studied.

## Introduction

Statistical studies of languages have become popular since the work of George Zipf [1] and have been refined with the availability of large data sets and the introduction of novel analytical models [2–7]. Zipf found that when words of large corpora are ranked according to their frequency, there seems to be a universal tendency across texts and languages. He proposed that ranked words follow a power law *f* ∼ 1/*k*, where *k* is the rank of the word—the higher ranks corresponding to the least frequent words—and *f* is the relative frequency of each word [8, 9]. This regularity of languages and other social and physical phenomena had been noticed beforehand, at least by Jean-Baptiste Estoup [10] and Felix Auerbach [11], but it is now known as Zipf’s law.

Zipf’s law is a rough approximation of the precise statistics of rank-frequency distributions of languages. As a consequence, several variations have been proposed [12–15]. We compared Zipf’s law with four other models, all of them behaving as 1/*k* ^*a*^ for a small *k*, with *a* ≈ 1, as detailed in the SI. We found that all models have systematic errors so it was difficult to choose one over the other.

Studies based on rank-frequency distributions of languages have proposed two word regimes [15, 16]: a “core” where the most common words occur, which behaves as 1/*k* ^*a*^ for small *k*, and another region for large *k*, which is identified by a change of exponent *a* in the distribution fit. Unfortunately, the point where exponent *a* changes varies widely across texts and languages, from 5000 [16] to 62,000 [15]. A recent study [17] measures the number of most frequent words which account for 75% of the Google books corpus. Differences of an order of magnitude across languages were obtained, from 2365 to 21077 words (including inflections of the same stems). This illustrates the variability of rank-frequency distributions. The core of human languages can be considered to be between 1500 and 3000 words (not counting different inflections of the same stems), based on basic vocabularies for foreigners [18], creole [19], and pidgin languages [20]. For example, Voice of America’s Special English [21] and Wikipedia in Simple English use about 1500 and 2000 words, respectively (not counting inflections). The Oxford Advanced Learner’s Dictionary lists 3000 priority lexical entries [22]. This suggests that the change of exponent *a* or another arbitrary cutoff in rank-frequency distributions does not reflect the size of the core of languages.

In view of these problems with rank-frequency distributions, we propose a novel measure to characterize statistical properties of languages. We have called this measure *rank diversity* and it tells us how words change their rank in time. With rank diversity, three regimes of words are identified: “heads”, “bodies” and “tails”. This measure of rank diversity follows the same simple functional law with similar parameters for all data analyzed. In particular, this is so for the six European languages studied here using a large data set of more than 6.4 ×10^11^ words from Google Books [23], which contains about 4% of all books written until 2008. It should be noted that this data set includes all different inflected forms (such as plural, different tense/aspect forms, etc.) found in the book corpus. Data sets such as this have allowed the study of “culturomics”: how cultural traits such as language have changed in time [24–30].

The rank diversity follows a scale-invariant behavior regarding its fluctuations, which inspires a model based on random walks, with scale-invariant random steps. This model reproduces the behavior of diversity and thus captures the essence of the evolution of word rank across different languages.

## Rank diversity of words

In what follows we shall consider six European languages from the Indo-European family. They are English and German; Spanish, French and Italian; and Russian. They belong to different linguistic branches: Germanic, Romance, and Slavic, respectively. The native speakers of these languages account for approximately 17% of the world population.

We shall start by taking into account the 20, say, most used words in the six languages, that is, the lowest-ranked words. Using, for the sake of uniformity, the first sense or first meaning given by Google Translate, once these words are translated into English, the coincidences in all six languages are remarkable (see Table S1 in S1 Text). This could have been foreseen, since most of the lowest-ranked words are articles, prepositions or conjunctions, *i.e.* what is called function words. A different matter, as we shall see, would result if we had considered only nouns, verbs, adverbs or adjectives, known as content words.

**Fig 1:**
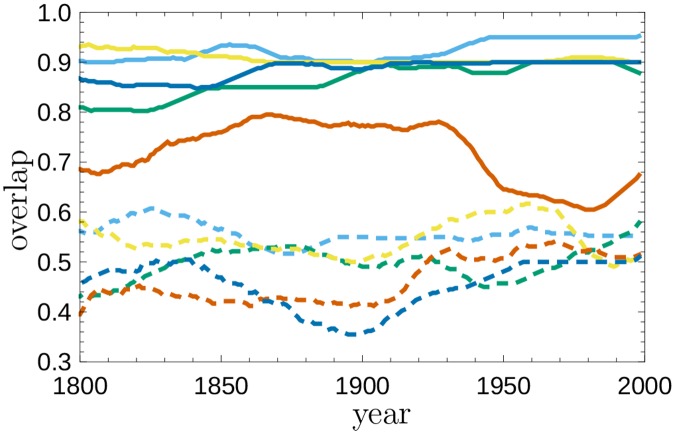
Overlap of the 20 most frequent words (continuous lines), and of the 20 most frequent *content* words (dashed lines) across languages, with respect to English, as a function of time. When words have more than one meaning, the first sense, according to Google Translate, was used. The color code for languages is as follows: light blue for French, green for German, yellow for Italian, dark blue for Spanish, and dark orange for Russian. Additionally, light orange will be used for English when required (see also Fig. 2). The same color coding for languages will be used throughout the rest of the article.

In order to quantify this fact, we present in Fig. 1 the time evolution of the overlap of the first 20 lowest-ranked words in the five languages with respect to the corresponding list of English. From the upper part of this figure we can see that along two centuries this overlap fluctuates around 0.9, a rather large number, except for Russian, since this language does not have articles. These data reveal that these Indo-European languages have shared structural properties, notwithstanding that they belong to distinct linguistic branches.

**Fig 2:**
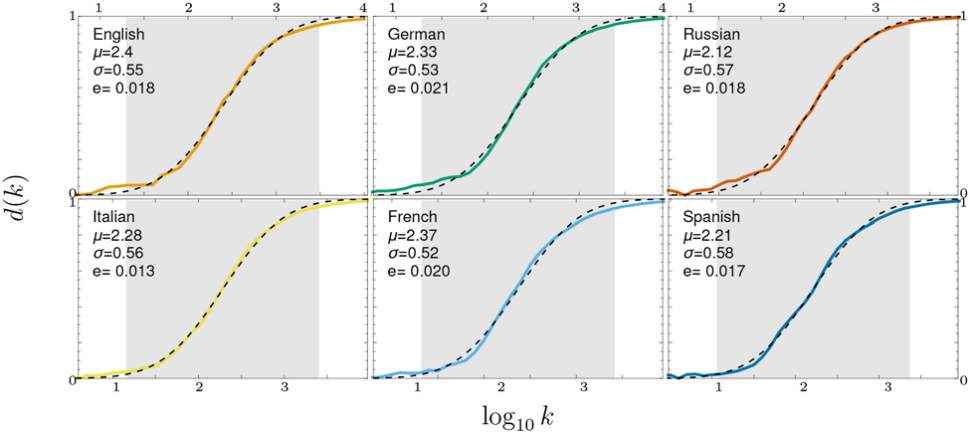
Rank diversity. Diversity *d* as a function of the rank *k* for different languages from 1800 to 2008, where *d*(*k*) measures how many different words appear for a given rank *k* during the time considered (Δ*t* = 208). For example, for English, *d*(1) = 1/208, as the word ‘the’ appears in the first rank for all years considered. Although we have analyzed up to *k* ≈ 10^6^, rank diversity for *k* > 10^4^ is not shown as *d*(*k*) ≈ 1, *i.e.*, a different word appears in each rank every year. Data are windowed over time, with a slot of size *δ*log_10_ *k* = 0.1, for the sake of clearness. Additionally, the sigmoid defined in Equation 1 is shown as a black dashed curve, with the best fit parameters, also reported in each subfigure. The mean square error *e* between the data and the fit, is also given. The shaded region corresponds to the average “body” of all languages.

The lowest-ranked words used to construct the upper part of Fig. 1 are essentially the same along centuries (See Figs S3-S8 in S1 Text). But this is not the case for content words, as can be seen in Table S2 in S1 Text. First, and as also shown by the dashed curves in Fig. 1, the overlap of these words with respect to English for the other five languages (including Russian) is of the order of 0.5. These values are much lower than the overlap of function words. Second, the most common nouns vary considerably with time. On the one hand, nouns like *time*, *man*, *life* and their translation to the other languages are present independently of the century. On the other hand, words like *god* and *king* have a low rank in the eighteenth century but have a larger rank in the last century. The rank change in time of these nouns reflect cultural facts.

What is discussed in the previous paragraph is an example of what could be called rank diversity *d*(*k*). This is, in the present study, the number of different words occurring at a specific rank *k* over a given period of time Δ*t*. We found that the resulting rank diversity curves for the six languages studied between 1800 and 2008 are similar to each other, as shown in Figs. 2 and 6. Low ranks have a very low diversity, as few words appear in the same ranks for the years we have studied.

As shown by the continuous lines in Fig. 2, the sigmoid curve fits very well *d*(*k*) for all languages considered, except for low *k* where the statistical fluctuations are larger due to the small sample size. The sigmoid is the cumulative of a Gaussian distribution, i.e.

$$\begin{array}{c} \Phi_{\mu ,\sigma }\left(\log_{10}k\right)=\frac{1}{\sigma \sqrt{2\pi }}\int_{-\infty }^{\log_{10}k}e^{-\frac{{\left(y-\mu \right)}^{2}}{2\sigma^{2}}}dy, \end{array}$$ (1)

and is given as a function of log *k*. The values of *μ* and *σ* reported in Fig. 2 were obtained adjusting Equation 1 to the rank diversity calculated for each individual language. The mean value *μ* identifies the point where *d*(*k*) ≈ 0.5, while the width *σ* gives the scale in which *d*(*k*) gets close to its extremal values. When log *k* is much larger than *μ*+*σ*, Φ_*μ*,*σ*_(log *k*) gets exponentially close to one, whereas when log *k* is much smaller than *μ*−*σ* it gets exponentially close to zero. It is customary in statistics to define a bulk of the Gaussian between *μ*±2*σ*, where 95% of the population lies. Along the same lines, we define three regions, marked by

$$\begin{array}{c} \log_{10}k_{\pm }=\mu \pm 2\sigma . \end{array}$$ (2)

**Fig 3:**
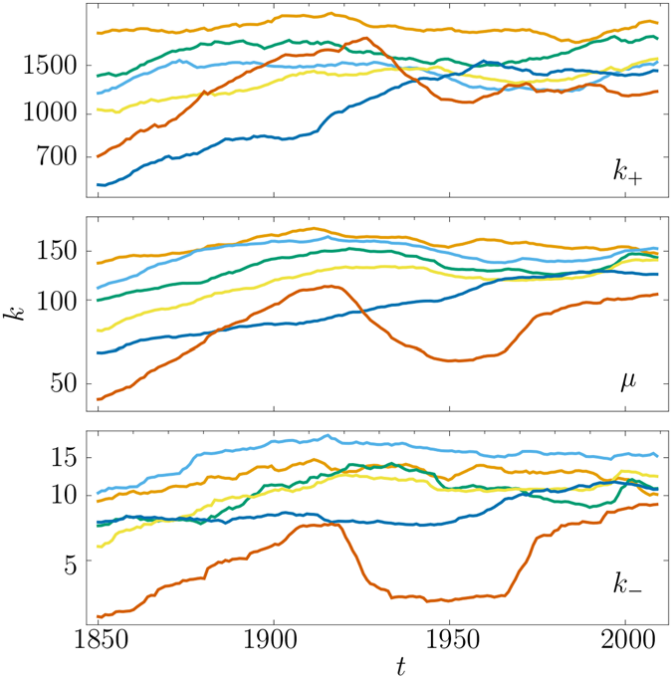
Evolution in time of the center of the sigmoid (middle panel), and the borders of the head and body (bottom panel) and body and tail (top panel) for the different languages along time for intervals of fifty years, *i.e.* Δ*t* = 50. Head words have *k* ≤ *k*_−_, body words have *k*_−_ < *k* ≤ *k*_+_, and tail words have *k*_+_ < *k*. See Fig. 2 for color coding.

First, we find what we shall call the head of the language, distributed with ranks between 1 and *k*_−_; a second region, identified as the body of the language, lies between *k*_−_ and *k*_+_; and finally the tail, beyond *k*_+_. From the values reported in Fig. 2, we see that 9 < *k*_−_ < 22, while *k*_+_ lies between 1832 and 3099. As shown in Fig. 3, these regions are robust to changes in the historical period considered and to the data set size (larger for recent years).

The bodies of languages consist of words that have limited change in time. Based on the size of basic vocabularies, it can be argued that the “core” of English is between 1500 and 3000 words, as mentioned in the introduction, which is consistent with our results. If we agree that the rank diversity identifies the core (head and body) of English, then it can be argued that the size of the core of the other five languages studied is similar [31], which is also supported by the high similarity across languages in Fig 2.

The tails of languages are formed by words which vary their rank considerably in time. This implies that they are more dependent on the text and its domain than words from the core. It can be assumed that words belonging to the head and body of languages have a high probability of being used in any text, while words from the tail would appear only in specific texts and domains.

Note that we obtain language cores slightly larger than those proposed by linguists. This is to be expected, as the Google Books data set treats words forms inflected for different persons, tenses, genders, numbers, cases, and so forth, as distinct items, while dictionaries count only stems (presented as citation forms, i.e. the basic form that users are most likely to look up). For example, the core for English obtained using rank diversity consists of 2448 words, but within these there are only 1760 different stems in the year 2008. Moreover, the studied data set contains several proper names which are not included in basic vocabulary lists. For English, 55 out of 2448 are proper names in 2008.

**Fig 4:**
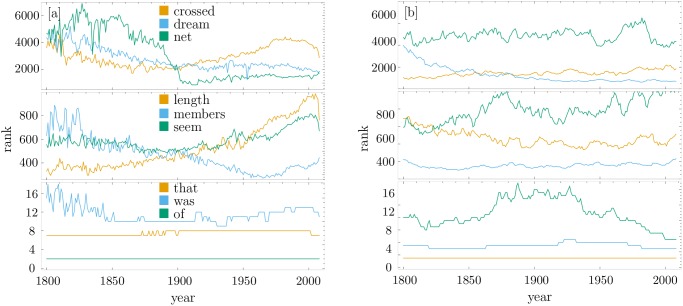
Rank evolution. [a]: Evolution of the rank for several particular, but random words in different regimes in the English language. From bottom to top we show words with initial ranks of order 1 (head), 100 (body) and 1000 (tail). [b]: Evolution of the rank for several particular, but random words in different regimes, for our scale-free Gaussian walker, i.e. the simulated language we have generated.

The rank evolution of particular words in time, belonging to the head, body, and tail of English is shown in Fig. 4a. This ratifies the results shown in Fig. 2, where low-ranked words exhibit little variation in time and this variation increases with the rank. More trajectories are presented in the SI. As mentioned above, words from the head vary little over time. However, the way in which words from the body or tail vary their rank in time appears to be similar, although at a different scale. This similarity leads us to propose a model of rank diversity where the amount of rank variation depends only on the rank.

## A random walk model for rank diversity

**Fig 5:**
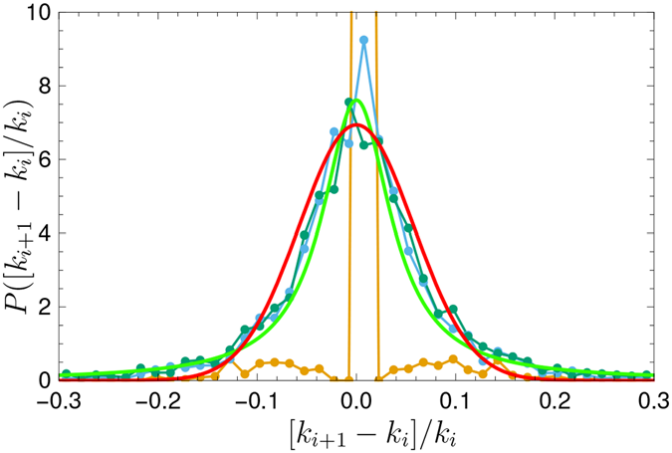
Distribution of relative size of frequency changes [*k*_*t*+1_−*k*_*t*_]/*k*_*t*_ in the case of English for words in the head (gold) (that start with rank between 1 and 10), the body (blue) (rank between 200 and 210), and the tail (green) (rank between 5000 and 5010). Notice that for words in the head, the granularity of the model (Equation 3) shows up as large deviations from the Gaussian. For the body and tail, the relative jumps are similar independently of the initial rank of the word. We also show, as a thick green curve, the Lorentzian distribution which best fits the average of the curves for the body and tail. A Gaussian, with zero mean and the most common standard deviation $\hat{\sigma }=0.0575$, is also shown in red for comparison (see text for details). The corresponding plot for other languages is shown in the supplementary information.

We consider the relative size of frequency changes, or flights as they are sometimes called in statistical physics, defined as (*k*_*t*+1_−*k*_*t*_)/*k*_*t*_ where *k*_*t*_ is the rank at discrete time *t* of a given element. We present in Fig. 5 the distribution of these frequency changes for English, our largest data set, and in Fig. S10 in S1 Text for all languages. Notice that, on average, the relative jumps seem to be largely independent of the value of the rank. We propose, based on this fact, a simple model to understand the evolution of rank diversity of words.

We shall call this model a scale-invariant random Gaussian walk, since a word with rank *k*_*t*_, is converted to rank *k*_*t*+1_ according to the following procedure: One defines an auxiliary variable *s*_*t*+1_ at time *t*+1 by the relation

$$\begin{array}{c} s_{t+1}=k_{t}+G\left(0,k_{t}\hat{\sigma }\right), \end{array}$$ (3)

where $G(0,\tilde{\sigma })$ is a Gaussian random number generator of width $\tilde{\sigma }$ and mean 0. This means that the random variable *s*_*t*+1_ has a width distribution proportional to *k*_*t*_. Words with very low ranks will change very slowly or not at all, while those with higher *k* have a larger rank variation in time, as reflected by *d*(*k*). Once the values of *s*_*t*+1_ for all words are obtained, they are ordered according to their magnitude. This new order gives new rankings, *i.e.* the *k* values at time *t*+1. There is a small correlation of the jumps between different times in this model. This is consistent with the observed behavior of the six languages dealt with here, as can be seen in Fig. S11 in S1 Text. The only parameter in the model is the width $\hat{\sigma }$, which is the most common standard deviation of the relative frequency changes of each data set.

A word of caution must be said. In Fig. 5, two curves are plotted. In green, a Lorentzian distribution, and in red a Gaussian distribution, both centered at zero, and with a width obtained by best fit to the data presented here. Although the Lorentzian fits these data somewhat better than the Gaussian, we use the latter in our model, since the long tails of the Lorentzian would yield long flights in words (not observed in the historical data) and a very different function *d*(*k*). One should recall that the Lorentzian does not have a finite second moment, so this might be the reason for this distribution to be inadequate. It is probable that a truncated Lorentzian could be a better choice, but we leave this detail open as a possible refinement to our model.

**Fig 6:**
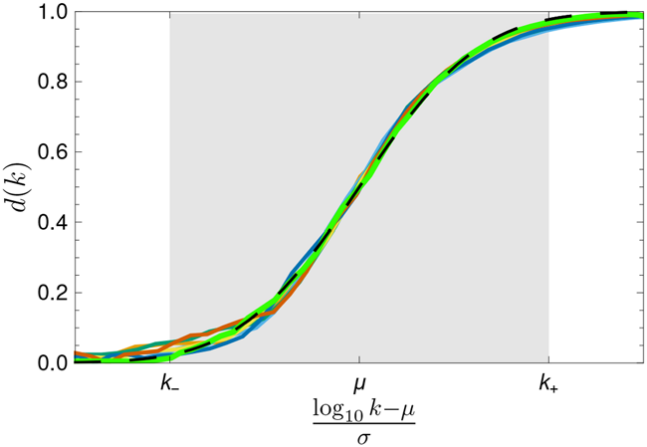
Rank diversity for the simulated language. The green curve represents the diversity corresponding to the language dynamics of a single realization of the Gaussian random walk model. We also include data for all languages studied, but normalized so that *k*_±_ coincide. The ansatz for the rank diversity is plotted as a parameter-free cumulative of a Gaussian with zero mean and unit variance as a dashed black curve.

With this model we have produced the evolution of a random simulated language; see [32] for other approaches. Fig. 4b shows examples of rank trajectories at different scales, exhibiting similarities with those of actual words shown in Fig. 4a. Moreover, if its diversity *d*(*k*) is calculated with the $\hat{\sigma }$ corresponding to the most popular width of the distribution of relative size of flights for all words in the English language from 1800 to 2008, the results coincide with the sigmoid obtained for all six languages analyzed, as shown in Fig. 6.

## Discussion

Within statistical linguistics, the frequency-rank distributions of several languages of European origin have been analyzed for many years now. However, no simple model can reproduce the detailed properties of this distribution (see SI). In particular, there has been the proposal that there exist two different regimes for ranks, but these regimes have not been satisfactorily validated in the empirical data. Due to these difficulties we have been led to introduce a statistical measure, which we have called rank diversity, to describe the statistical properties of natural languages. A simulated random language was generated which reproduces the observed features quite well.

Our random walk model mimics the evolution of languages to produce a simulated rank diversity which closely matches that of historical data. We consider that statistical similarities across languages and the simplicity of the model to reproduce them sufficient evidence to claim that rank diversity of words is universal. This does not imply that all languages have the same rank diversity curves, but that the rank diversity distribution of all the languages studied here can be fitted properly with Equation 1. Certainly, different languages have different curves that fit them better, just as different exponents fit better a Zipf distribution of different languages. For the languages studied, 1.6 ≤ *μ* ≤ 2.1 and 0.4 ≤ *σ* ≤ 0.6.

This universality could be used to favor nativist explanations of human language [33, 34], where language is claimed to be determined by innate constraints. However, the high-ranked diversity of language tails could be used in favor of adaptationist explanations as well [35], as the precise rank of tail words is highly contingent. In recent years, explanations of human language relating biological evolution (genetically encoded innate properties) and learning (epigenetical adaptation) with culture have gained strength [36–38]. Even so, few assumptions are necessary to explain some general aspects of the evolution of human languages [39]. The present work shows that the evolution of word frequency can be explained with Gaussian random walks, where the size of the change in word frequency is proportional to its rank, *i.e.* frequent words change less than infrequent words. This explanation does not require innate properties, adaptive advantages, nor culture. This does not imply that the latter are irrelevant for other aspects of language evolution. Note that our study is carried out at a statistical level. We do not address syntactic, semantic, and grammatical aspects of human language [40–43], which are certainly important.

Why does the rank diversity approach a lognormal distribution? Which processes and mechanisms are required for this? There is one condition for a variable to have a lognormal distribution. This condition is that the variable should be the result of a high number of different and independent causes which produce positive effects composed multiplicatively. Thus, each cause has a negligible effect on the global result [44]. Our Gaussian random walk model supports this as a suitable explanation: the statistical distribution of *d* is always lognormal, there is a high number of components (words), each word has a negligible effect compared to the language properties, *i.e.* large changes in word frequency (ranking) do not cause large changes in the statistical properties of each language, and the rank of each word is partially a cumulative product of its rank in previous times, as expressed in Equation 3. Languages statistically comply with these dynamics, and that serves as an explanation for their evolution and structure.

**Fig 7:**
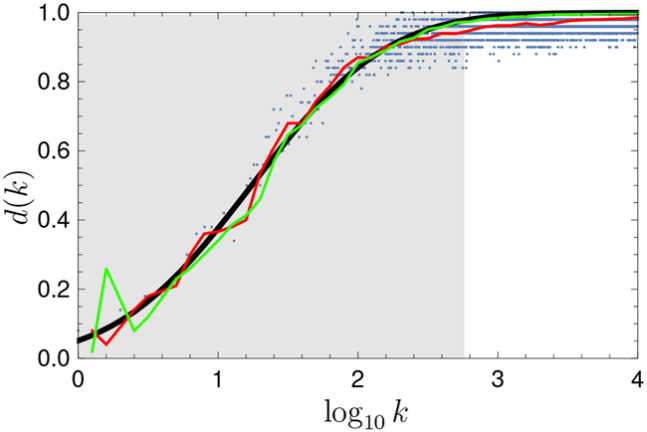
Rank diversity of male chess players obtained from the trimestral FIDE rankings from April, 2001 to May, 2012 (Δ*t* = 50), considering the first 10,000 ranks. Blue dots show rank diversity, windowed in the red line. The black line shows the sigmoid fit with *μ* = 1.24 and *σ* = 0.76. The green line shows a simulation with $\hat{\sigma }=0.18$. Notice that there is no head as *μ*−2*σ* < 0. This is to be expected, as many players enter and leave the ranking during the years considered.

In future work, it will be relevant to study the rank diversity of *n*-grams with *n* > 1 [45], other linguistic corpora and phenomena with dynamic rank distributions [27, 46–48] and more generally with temporal networks [49–52]. A specific example would be the ranking of chess players, given by the World Chess Federation (Fédération Internationale des Échecs). The rank diversity in this case is provided in Fig. 7, which shows that the sigmoid is appropriate also for this case.

## Supporting Information

S1 Text **Figure S1. Rank distributions of words according to frequency.** [a]: Normalized word frequency *f*_R_ as a function of the rank *k* for several languages for books published in the year 2000. The color code for languages is as follows: light blue for French, green for German, yellow for Italian, orange for English, dark blue for Spanish, and red for Russian. [b]: Word frequency *f*_R_ as a function of the rank *k* for English and several years, normalized so that the most frequent element has relative frequency one. In the inset, the unnormalized frequency *f* is shown.
**Figure S2. Comparison between the different models, Equations S1–S5, and the frequency of rank distribution.** We use the data for the year 2000 and all languages under consideration. The logarithm base 10 of the ratio of the observed values and the model is plotted. It can be appreciated that different models fit better in different regions. However there is no model that fits all languages and all regions much better than the others.
**Figure S3. Rank variations in time of twenty words from three different scales for English.**
**Figure S4. Rank variations in time of twenty words from three different scales for German.**
**Figure S5. Rank variations in time of twenty words from three different scales for French.**
**Figure S6. Rank variations in time of twenty words from three different scales for Italian.**
**Figure S7. Rank variations in time of twenty words from three different scales for Spanish.**
**Figure S8. Rank variations in time of twenty words from three different scales for Russian.**
**Figure S9. Rank variations in time of twenty words from three different scales for our simulated language.**
**Figure S10. Distribution of relative flights for all languages studied.** A similar plot as the one presented in Fig. 5 is shown for other languages. The same color coding and details are used.
**Figure S11. Correlations for relative frequency changes** for different languages. Black line shows correlations for simulated language.
(PDF)
