# Supplementary material for: Rank Diversity of Languages: Generic Behavior in Computational Linguistics
Source: PLoS One. 2015 Apr 7;10(4):e0121898. doi: 10.1371/journal.pone.0121898 (PMC4388647; doi:10.1371/journal.pone.0121898)
Supplement: S1 Text — Figure S1. Rank distributions of words according to frequency. [a]: Normalized word frequency f R as a function of the rank k for several languages for books published in the year 2000. The color code for languages is as follows: light blue for French, green for German, yellow for Italian, orange for English, dark blue for Spanish, and red for Russian. [b]: Word frequency f R as a function of the rank k for English and several years, normalized so that the most frequent element has relative frequency one. In the inset, the unnormalized frequency f is shown. Figure S2. Comparison between the different models, Equations S1–S5, and the frequency of rank distribution. We use the data for the year 2000 and all languages under consideration. The logarithm base 10 of the ratio of the observed values and the model is plotted. It can be appreciated that different models fit better in different regions. However there is no model that fits all languages and all regions much better than the others. Figure S3. Rank variations in time of twenty words from three different scales for English. Figure S4. Rank variations in time of twenty words from three different scales for German. Figure S5. Rank variations in time of twenty words from three different scales for French. Figure S6. Rank variations in time of twenty words from three different scales for Italian. Figure S7. Rank variations in time of twenty words from three different scales for Spanish. Figure S8. Rank variations in time of twenty words from three different scales for Russian. Figure S9. Rank variations in time of twenty words from three different scales for our simulated language. Figure S10. Distribution of relative flights for all languages studied. A similar plot as the one presented in Fig. 5 is shown for other languages. The same color coding and details are used. Figure S11. Correlations for relative frequency changes for different languages. Black line shows correlations for simulated language. (PDF) [file pone.0121898.s001.pdf]

Supplementary Information: Rank diversity of languages: Generic behavior in computational linguistics

Germinal Cocho, Jorge Flores,
Carlos Gershenson, Carlos Pineda, Sergio Sánchez

January 8, 2015

S1 Models for rank-frequency distributions

The rank-frequency distributions of words for different languages are very similar to each other, as shown in Fig. S1a. The distributions are also similar across centuries, as shown in Fig. S1b.

We present five different distributions with distinct origins, though all of them containing the common factor $\frac{1}{k^a}$. The distributions are:

$$m_1(k) = \mathcal{N}_1 \frac{1}{k^a}, \tag{S1}$$

$$m_2(k) = \mathcal{N}_2 \frac{\mathrm{e}^{-b(k-1)}}{k^a}, \tag{S2}$$

$$m_3(k) = \mathcal{N}_3 \frac{(\bar{N}+1-k)^\alpha}{k^a}, \tag{S3}$$

$$m_4(k) = \mathcal{N}_4 \frac{(\bar{N}+1-k)^\alpha \mathrm{e}^{-b(k-1)}}{k^a}, \tag{S4}$$

$$m_5(k) = \mathcal{N}_5 \begin{cases} \frac{1}{k} & k \le k_c \\ \frac{k_c^{a-1}}{k^a} & k > k_c \end{cases} \tag{S5}$$

where $\mathcal{N}_i$ are normalization factors, depending on the parameters a, b, and α of the different models, and $\bar{N}$ is the total number of words.

In Fig. S2 we compare the fit of these distributions with the observed curves. It can be seen that none of the distributions reproduces closely the dataset. We calculated for all fits the χ^2 test with similar results. The best value corresponds to the fit proposed in [1], namely the double Zipf model (equation (S5)). In all cases we studied the p-value of the data, needed for an appropriate interpretation of the goodness of the fit. In all cases, that is for all years, all languages and all models, this number was smaller than machine precision. This shows that none of these models captures satisfactorily the data behavior.

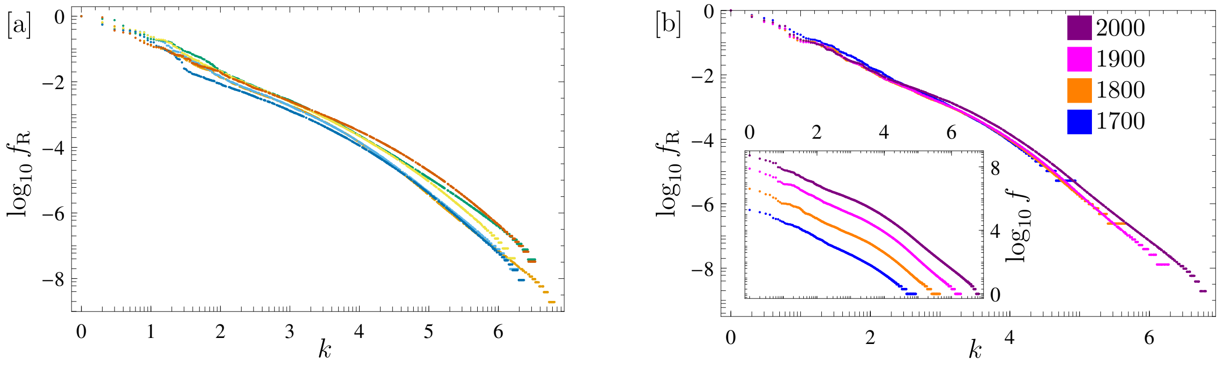

Figure S1: Rank distributions of words according to frequency. [a]: Normalized word frequency f_R as a function of the rank k for several languages for books published in the year 2000. The color code for languages is as follows: ■ for French, ■ for German, ■ for Italian, ■ for English, ■ for Spanish, and ■ for Russian. [b]: Word frequency f_R as a function of the rank k for English and several years, normalized so that the most frequent element has relative frequency one. In the inset, the unnormalized frequency f is shown.

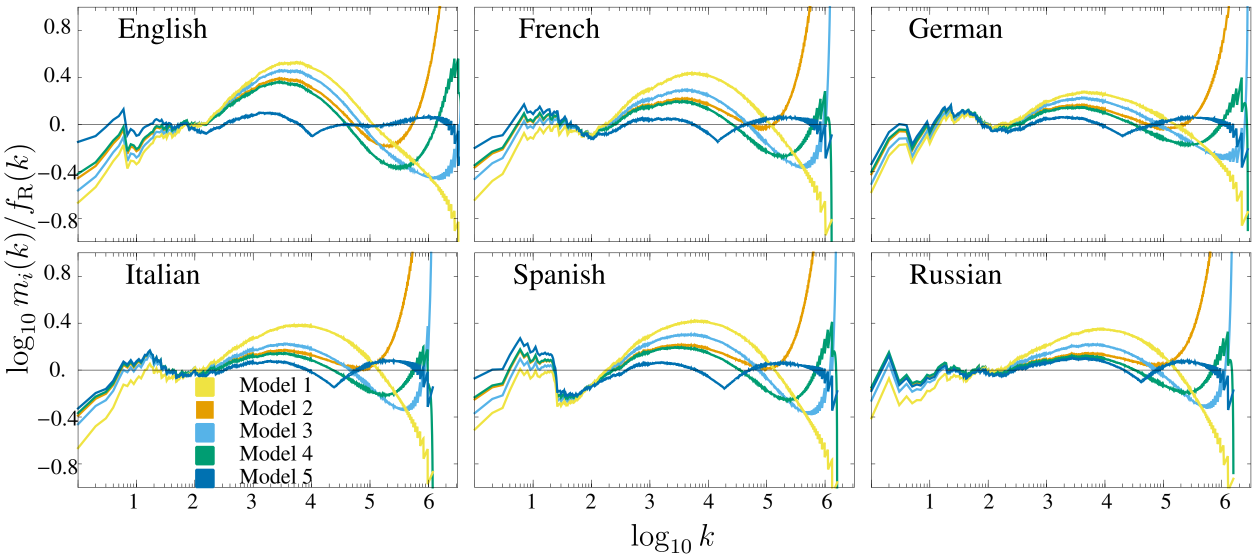

Figure S2: Comparison between the different models, equation (S1)–equation (S5), and the frequency of rank distribution. We use the data for the year 2000 and all languages under consideration. The logarithm base 10 of the ratio of the observed values and the model is plotted. It can be appreciate that different models fit better in different regions. However there is no model that fits all languages and all regions much better than the others.

The origin of some of these models is similar. The following discussion shows how they can be encompassed in a common formulation.

Given a set of words forming a text, one can evaluate the number of times $N(k,t)$ that a certain word appears with the rank k at time t. If $B(k)$and $D(k)$ denote, respectively, the probability per unit time that a word enters or leaves the rank k, we have:

$$\frac{\partial}{\partial t}N(k,t) = \{\xi(k) - F[\Sigma(t)]\}\, N(k,t) \\ + \Big\{D(k+1)N(k+1,t) + B(k-1)N(k-1,t) - [D(k)+B(k)]\, N(k,t)\Big\}. \quad \text{(S6)}$$

Here the two terms on the r.h.s. within the first curly brackets describe, respectively, the local growth rate and the overall decrease rate acting on $N(k,t)$. The total number of words at a given time t is $\Sigma(t)$ and F is a function that determines global constraint features that refer to the total number of words. The terms within the second curly brackets, indicate the balance arising from the birth $B(k)$ and death $D(k)$ contributions of first neighbor words with $k \pm 1$ ranks at time t. If we consider the total number of words at a given time t to be a fixed quantity,

$$\Sigma(t) = \sum_k N(k,t) \quad \text{(S7)}$$

we can define the probability density of finding a word with rank k, or relative frequency distribution, by

$$n(k,t) \equiv \frac{N(k,t)}{\Sigma(t)}. \quad \text{(S8)}$$

Substitution of equation (S7) and equation (S8) into equation (S6) leads to

$$\frac{d}{dt}\Sigma(t) = \langle \xi(k) \rangle - F[\Sigma(t)]\Sigma(t), \quad \text{(S9)}$$

where the bracket indicates a sum over all k weighted by $n(k,t)$. We assume, for simplicity, that $\langle \xi(k) \rangle$ is a linear function of the number of edges k, so that $\langle \xi(k) \rangle = \xi_0 + \xi_1 \langle k \rangle$, where ξ_0 and ξ_1 are constants. Then equation (S6) reduces to the following master equation for a one step process:

$$\dot{n}(k,t) = V(k)n(k,t) \\ + \{D(k+1)n(k+1,t) + B(k-1)n(k-1,t) - [D(k)+B(k)]\, n(k,t)\}\,, \quad \text{(S10)}$$

where $\dot{n}(k,t) \equiv \partial n(k,t)/\partial t$ and the effective potential $V(k) \equiv \xi(k) - \langle \xi(k) \rangle$ has the property

$$\langle V(k) \rangle = 0. \quad \text{(S11)}$$

In what follows we shall only consider the case $\xi(k) = \xi_0$, so equation (S10) reduces to the general form of the master equation for a one step process,

$$\dot{n}\,(k,t) = D\,(k+1)\,n\,(k+1,t) + B\,(k-1)\,n\,(k-1,t) - [D(k)+B(k)]\,n(k,t). \quad \text{(S12)}$$

If the changes in k are small and we are only interested in solutions $n(k,t)$ that vary slowly with k, then k may be treated as a continuous variable and we obtain the Fokker-Planck equation:

$$\frac{\partial n(k,t)}{\partial t} = -\frac{\partial}{\partial k}\left[g(k)\, n(k,t)\right] + \frac{1}{2}\frac{\partial^2}{\partial k^2}\left[f(k)\, n(k,t)\right], \tag{S13}$$

where $f(k) = B(k) + D(k)$ and $g(k) = B(k) - D(k)$. For the stationary solutions $m(k)$, we have the equation

$$g(k)m(k) = \frac{1}{2}\frac{d}{dk}\left[f(k)m(k)\right]. \tag{S14}$$

If we approximate $g(k)/f(k)$ by Padé approximants $g_n(k)/f_n(k) = A_0 + \sum_{k=1}^{n} \frac{A_k}{(k+c_k)}$, the stationary solution becomes

$$m(k) = \mathcal{N} \exp\left(A_0 k\right) \prod_{k=1}^{n} (k + c_k)^{-A_k}. \tag{S15}$$

If we assume the simplest expression for $D(k)$ and $B(k)$ transition probabilities

$$D(k) = \lambda_1 (c_1 + k)(N_1 - k), \tag{S16}$$

$$B(k) = \lambda_2 (c_2 + k)(N_2 - k) \tag{S17}$$

then

$$m(k) = \mathcal{N} \exp(A_0 k) \frac{\left(\bar{N} - k\right)^b}{(\bar{c} + k)^a}, \tag{S18}$$

where $\bar{N} = \frac{1}{2}(N_1 + N_2)$, $\bar{c} = \frac{1}{2}(c_1 + c_2) = 1$, $a = c_1 - c_2 + 1$, and $b = N_1 - N_2 - 1$. Also we must remember that in our case k starts at one. Then if $A_0 = b = 0$, we have the Zipf model; when $A_0 \neq 0$ and $b = 0$, the γ model is gotten (equation (S2)); if $A_0 = 0$ but $b \neq 0$ the β model is obtained (equation (S3)); finally, if A_0 and b are different from 0, we have the general $\beta\gamma$ model (equation (S4)).

These and additional results could be obtained using the complex network language [2, 3, 4].

With respect to the distribution of equation (S5), the derivation given in [1] is based on the following assumptions. The existence of two word regimes: A language core containing words with low rank and do not affect the birth of new words, and the remaining high ranked words which reduce the probability of new words to be used.

S2 Variation of words in time

Table S1 shows the most frequent words for the year 2000 with their translation and relative frequency. Notice that these are very similar across languages. Table S2 shows the most frequent nouns for the years 1700, 1800, 1900, and 2000. There are similarities across languages and across centuries, but also important differences.

Table S1: Lowest–rank words for several languages in books published during the year 2000, together with their translation to English and their relative frequency.

rank	English	German	French	Italian	Spanish	Russian
1	the, 0.065530	der, the, 0.038512	de, of, 0.057225	di, of, 0.041518	de, of, 0.073063	и, and, 0.053961
2	of, 0.036769	die, the, 0.036010	la, the, 0.035222	e, and, 0.028107	la, the, 0.043297	в, in, 0.053922
3	and, 0.029289	und, and, 0.028087	et, and, 0.024466	la, the, 0.020308	en, in, 0.029059	на, on, 0.020190
4	to, 0.025264	in, in, 0.020607	le, the, 0.022384	che, that, 0.017861	y, and, 0.028908	не, not, 0.017334
5	in, 0.021769	von, of, 0.011277	les, the, 0.021076	il, the, 0.017702	el, the, 0.027771	что, what, 0.011770
6	a, 0.020715	den, the, 0.011012	à, to, 0.019951	in, in, 0.017357	que, that, 0.026713	по, by, 0.010202
7	is, 0.010712	zu, to, 0.010488	des, of, 0.019212	a, to, 0.014067	a, to, 0.019706	к, to, 0.008559
8	that, 0.010529	des, of, 0.010102	en, in, 0.014334	del, of, 0.013403	los, the, 0.018039	как, as, 0.008027
9	for, 0.008975	das, the, 0.009806	du, of, 0.012991	della, of, 0.010876	del, of, 0.013492	а, and, 0.007745
10	as, 0.007396	im, in the, 0.007418	un, a, 0.011112	per, for, 0.010480	se, oneself, 0.012448	о, about, 0.006824
11	it, 0.006832	mit, with, 0.007403	une, a, 0.010825	un, a, 0.009949	las, the, 0.012294	из, of, 0.006356
12	with, 0.006707	sich, itself, 0.007337	dans, in, 0.010145	non, not, 0.008645	por, by, 0.009908	его, his, 0.005911
13	was, 0.006576	ist, is, 0.007197	que, that, 0.009896	si, oneself, 0.008515	un, a, 0.008824	для, for, 0.005822
14	on, 0.006289	auf, on, 0.007047	qui, who, 0.008609	è, is, 0.008501	con, with, 0.008469	от, from, 0.005769
15	not, 0.005970	nicht, not, 0.006875	par, by, 0.007494	una, a, 0.007891	una, a, 0.007863	он, he, 0.005538
16	be, 0.005671	für, for, 0.006874	est, is, 0.007258	le, the, 0.007852	no, no, 0.007547	но, but, 0.005324
17	by, 0.005440	eine, a, 0.006757	pour, for, 0.007027	i, the, 0.007626	para, for, 0.006877	я, I, 0.005097
18	i, 0.005212	als, as, 0.006521	il, it, 0.006749	con, with, 0.006734	su, its, 0.006597	это, this, 0.004925
19	are, 0.004928	dem, the, 0.005723	au, to the, 0.006429	da, from, 0.006258	es, is, 0.006086	за, for, 0.004623
20	this, 0.004916	auch, also, 0.005630	a, has, 0.005504	nel, in, 0.005184	al, to the, 0.005855	у, at, 0.003862

Figs. S3–S9 show rank trajectories of words for the languages studied, including our simulated language. It can be seen that the behavior is similar for all languages: words with low rank (heads) almost do not vary in time. Afterwards the variation in rank depends on the rank itself, approximating a scale-invariant random walk. Notice that there is a higher variation at all scales before 1850. Further work is required to measure how much this variation depends on having less data before 1850 and how much on language properties of the time.

Fig. S10 shows the distribution of relative flights for all languages. See main text for details.

S3 Correlation of relative frequency changes

We studied the correlations of the relative frequency changes (flights), defined in the main text as

$$\Delta_t = (k_{t+1} - k_t)/k_t. \tag{S19}$$

We shall use a normalized version of it:

$$d_t = \frac{\Delta_t - \langle \Delta_t \rangle}{\sqrt{\langle (\Delta_t - \langle \Delta_t \rangle)^2 \rangle}}, \tag{S20}$$

where $\langle \cdot \rangle$ denotes average over time. This normalization ensures that both $\langle d_t \rangle = 0$ and $\langle d_t^2 \rangle = 1$. The time correlation is given by

$$C_\tau = \langle d_t d_{t+\tau} \rangle. \tag{S21}$$

In principle, this quantity also depends on t, but usually this dependence is very weak, as in this case, and one can ignore it.

In Fig. S11 we show the average of C_τ, of 50 different ranks chosen randomly, for different languages, as well as for the simulated language. We note that the correlation is very small, except for $\tau = 0$, where it is 1, due to the normalization chosen, and for $\tau = 1$ where a negative value, typical of bounded sequences, is observed for the six languages studied here. The random Gaussian model reproduces well these correlations except at $\tau = 1$.

References

[1] Gerlach M, Altmann EG (2013) Stochastic model for the vocabulary growth in natural languages. Phys Rev X 3: 021006.

[2] Albert R, Barabási AL (2002) Statistical mechanics of complex networks. Rev Mod Phys 74: 47–97.

[3] Jensen HJ (2008) Emergence of network structure in models of collective evolution and evolutionary dynamics. Proceedings of the Royal Society A: Mathematical, Physical and Engineering Science 464: 2207-2217.

[4] McKane A, Alonso D, Solé RV (2000) Mean-field stochastic theory for species-rich assembled communities. Phys Rev E 62: 8466–8484.

Table S2: Lowest ranked **nouns** for different years (top left cell) and different languages. Note that some words are used not only as nouns, which can give them a higher rank. For example, *été* in French is summer, but also the past participle of *être* (to be).

1700	English	German	French	Italian	Spanish	Russian
1	god	Erfahrung, experience	fait, fact	rei, king	fe, faith	день, day
2	man	Gottesfurcht, fear of god	dieu, god	sez, section	señor, mr.	города, city
3	men	Derselben, the same	point, point	civ, civil code	cardenal, cardinal	капитанъ, captain
4	people	Denselben, the same	corps, body	giudice, judge	rey, king	года, year
5	first	Dieselbe, the same	amour, love	parte, part	dios, god	утру, morning
6	things	Dieselben, the same	car, car	comma, paragraph	solo, single	полки, shelves
7	time	Denselben, the same	Reims, Reims	lavoro, work	tiempo, time	ночь, night
8	world	Menschen, people	temps, time	diritto, right	san, saint	лошадей, horses
9	thing	Alter, age	homme, man	art, article	duque, duke	городъ, city
10	power	Jugend, youth	roy, king	sentenza, judgment	ácido, acid	вечеру, evening
1800	English	German	French	Italian	Spanish	Russian
1	time	Nichts, nothing	fait, fact	era, era	dios, god	время, time
2	king	Zeit, time	point, point	parte, part	parte, part	году, year
3	man	Art, type	été, summer	tempo, time	tiempo, time	день, day
4	god	Derselben, the same	eau, water	prima, first	solo, single	года, year
5	first	Menschen, people	partie, part	stato, state	señor, mr.	времени, time
6	part	Allein, alone	corps, body	città, city	hombre, man	людей, people
7	men	Natur, nature	temps, time	repubblica, republic	cuerpo, body	города, city
8	general	—	terre, land	cose, things	vida, life	образомъ, way
9	people	—	nombre, number	fatto, fact	modo, mode	земли, land
10	place	—	homme, man	luogo, place	hombres, men	будетъ, will
1900	English	German	French	Italian	Spanish	Russian
1	time	Selbst, even	été, summer	era, era	señor, mr.	время, time
2	man	Jahre, years	fait, fact	parte, part	parte, part	года, year
3	first	Weise, wise	point, point	stato, state	ley, law	жизни, life
4	life	Ersten, first	temps, time	legge, law	gobierno, government	времени, time
5	men	Recht, right	cas, case	prima, first	estado, state	образомъ, way
6	day	Art, type	droit, right	fatto, fact	derecho, right	будетъ, will
7	old	Einzelnen, individual	loi, law	tempo, time	años, years	томъ, volume
8	years	Frage, question	partie, part	vita, life	año, year	году, year
9	work	Nichts, nothing	Paris, Paris	anni, age	ciudad, city	права, right
10	people	—	France, France	Italia, Italy	artículo, article	право, right
2000	English	German	French	Italian	Spanish	Russian
1	time	Deutschen, German	fait, fact	era, era	parte, part	время, time
2	first	Jahre, years	été, summer	parte, part	años, years old	том, volume
3	people	Menschen, people	paris, Paris	stato, state	estado, state	года, year
4	work	Frage, question	temps, time	prima, first	vida, life	федерации, federation
5	way	Deutschland, Germany	pays, country	anni, years	años, years	жизни, life
6	life	Jahren, years	politique, policy	vita, life	nacional, national	лет, years
7	world	Berlin, Berlin	vie, life	tempo, time	tiempo, time	человек, man
8	way	Ersten, first	france, France	secondo, second	social, social	году, year
9	state	Entwicklung, development	travail, work	modo, way	forma, form	раз, time
10	years	Arbeit, work	monde, world	fatto, fact	polı́tica, policy	человека, human

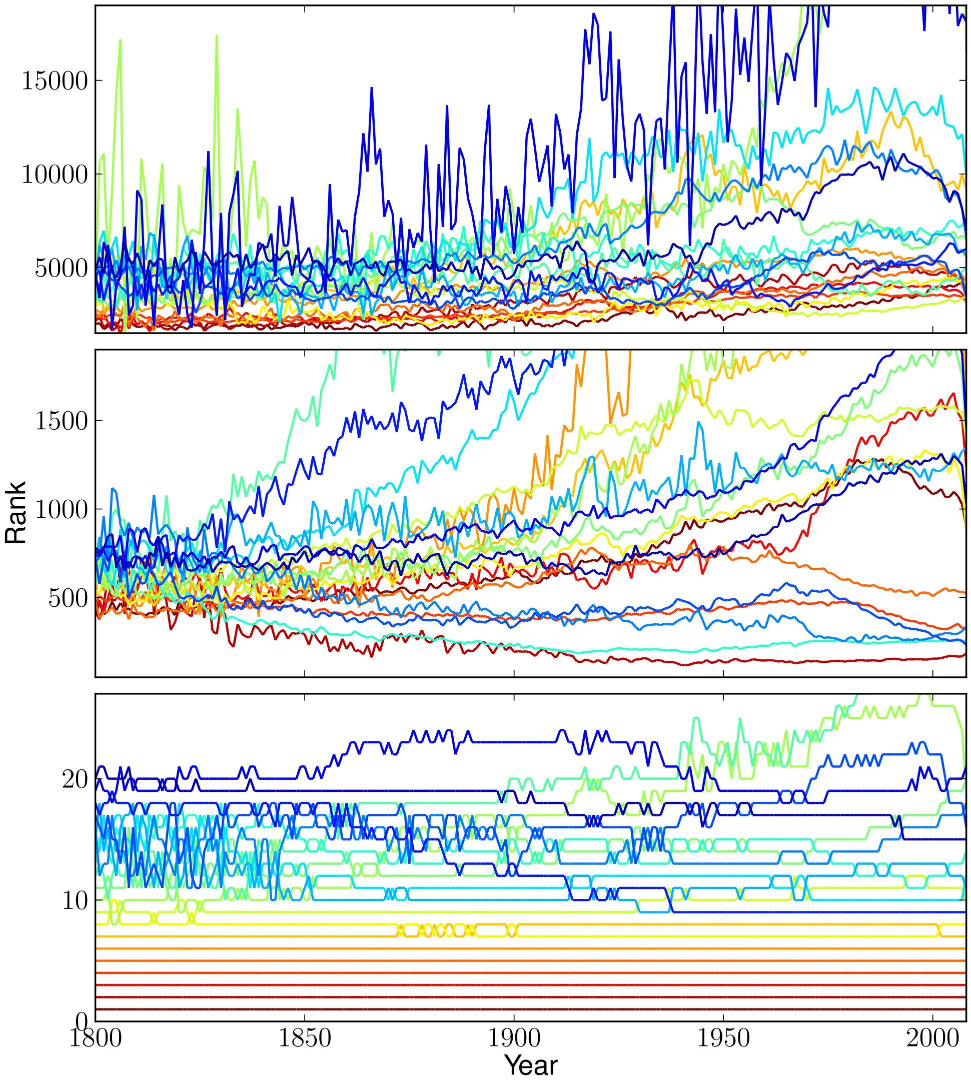

Figure S3: Rank variations in time of twenty words from three different scales for English.

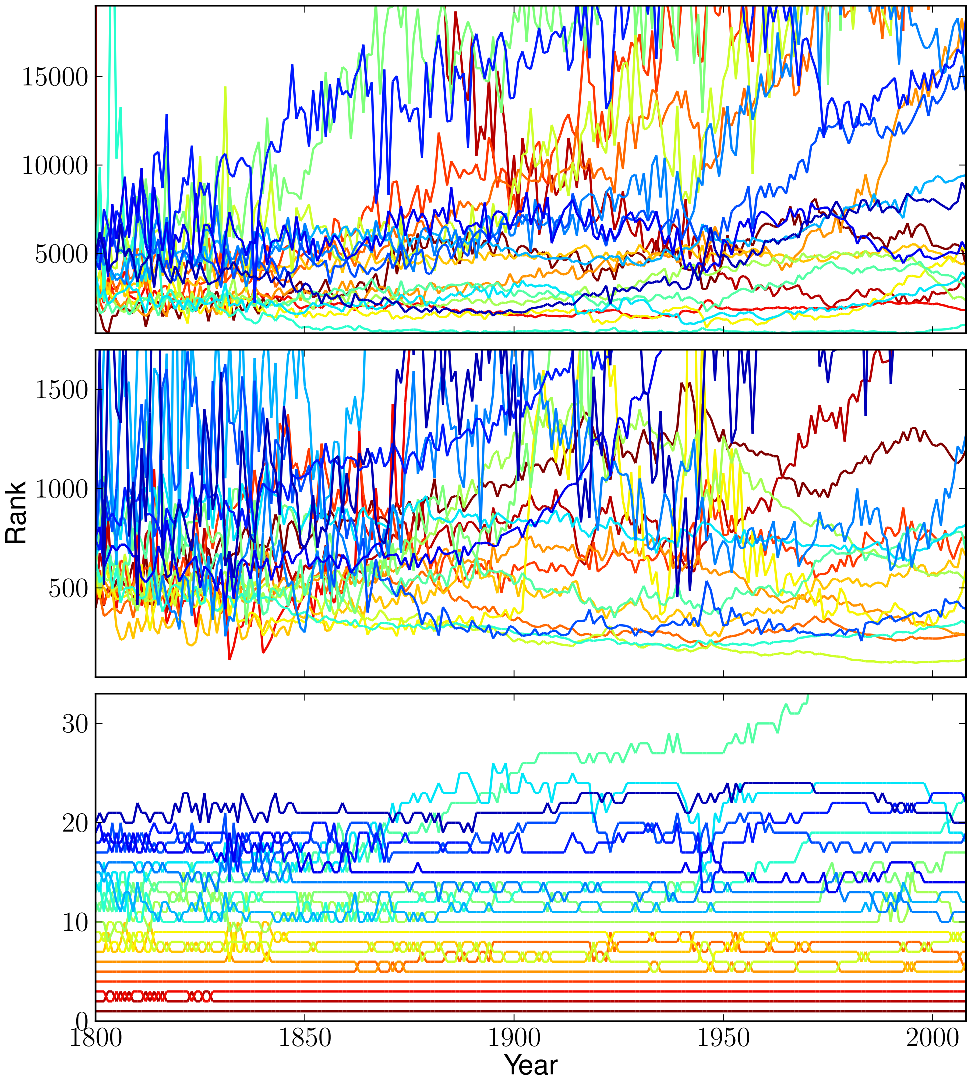

Figure S4: Rank variations in time of twenty words from three different scales for German.

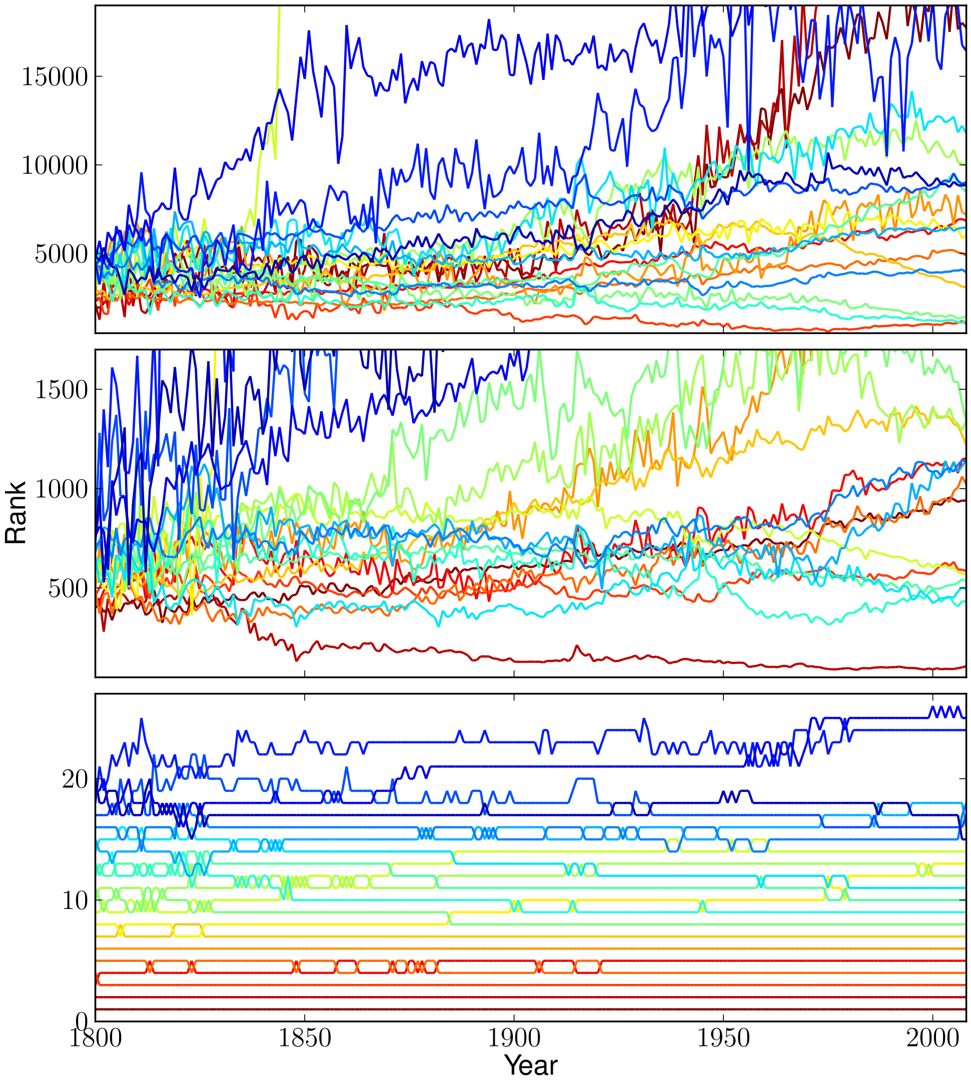

Figure S5: Rank variations in time of twenty words from three different scales for French.

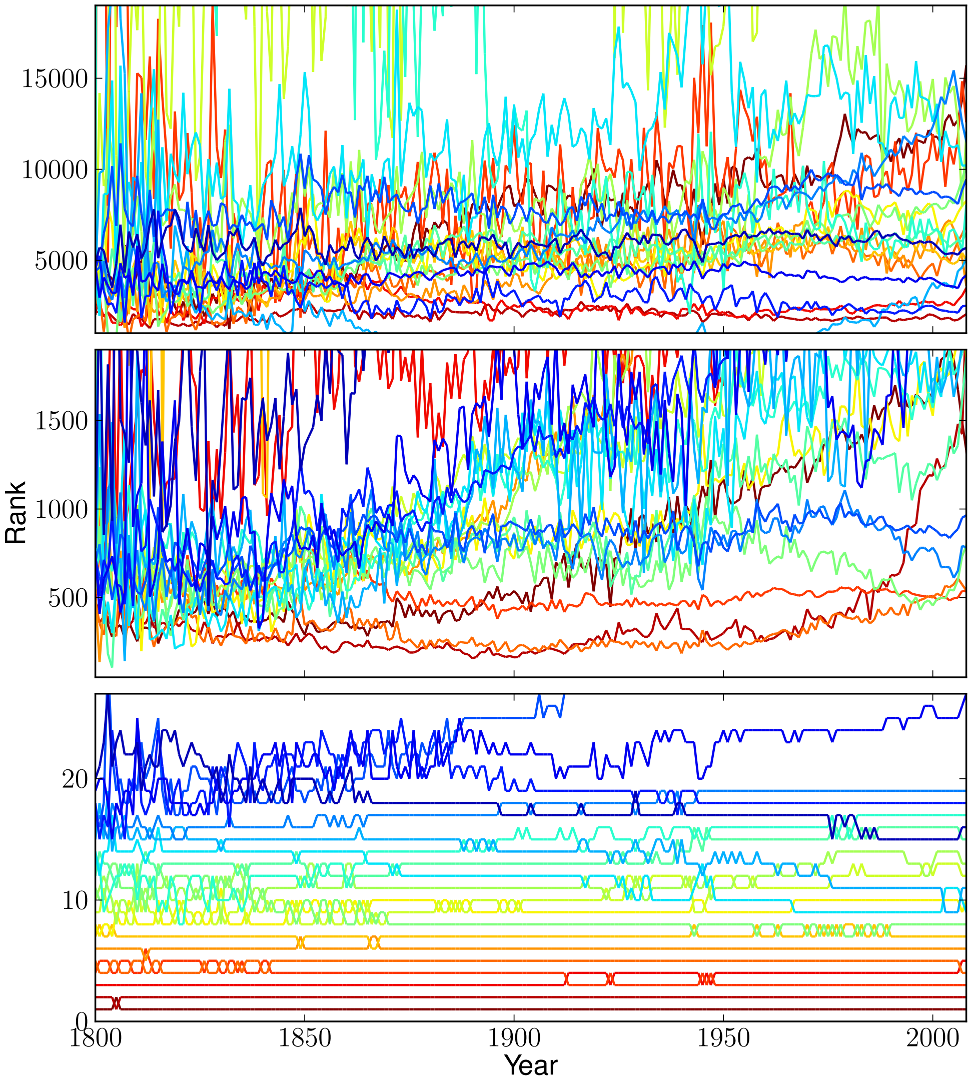

Figure S6: Rank variations in time of twenty words from three different scales for Italian.

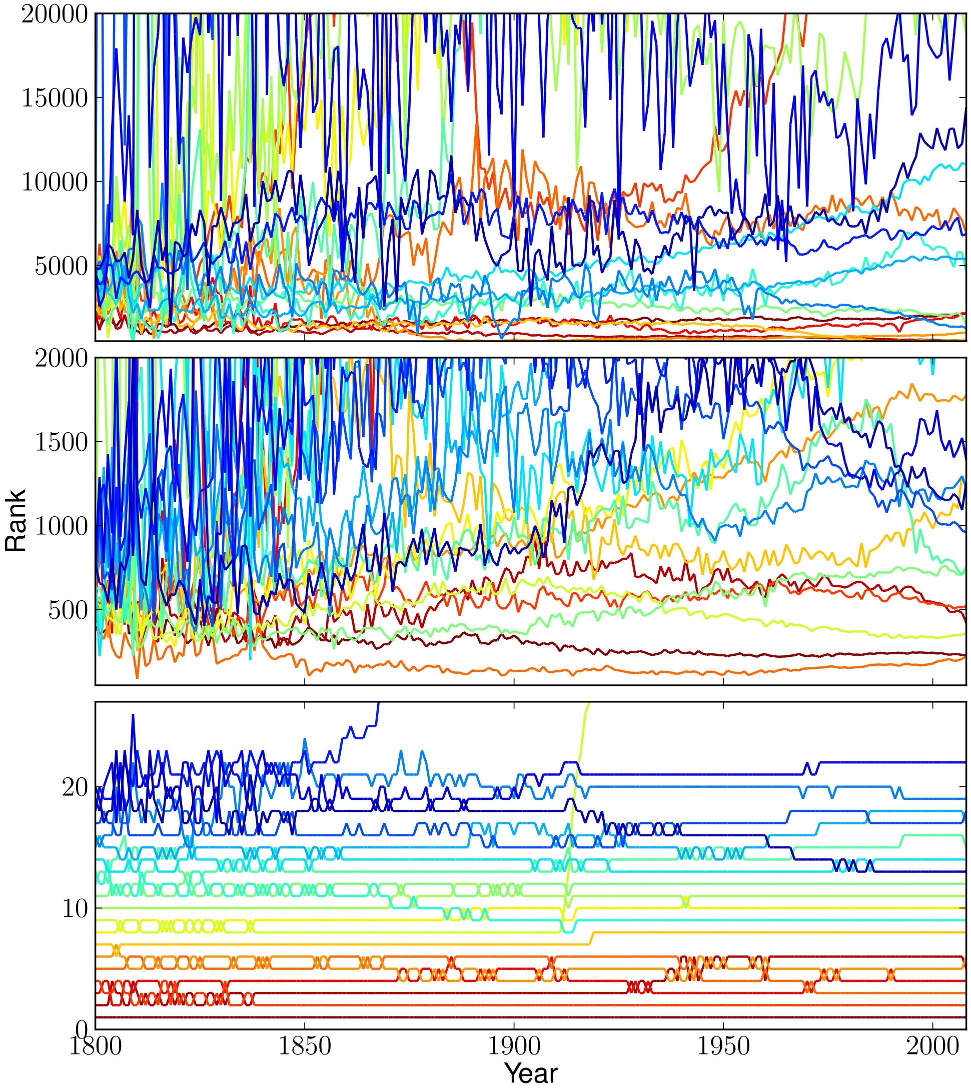

Figure S7: Rank variations in time of twenty words from three different scales for Spanish.

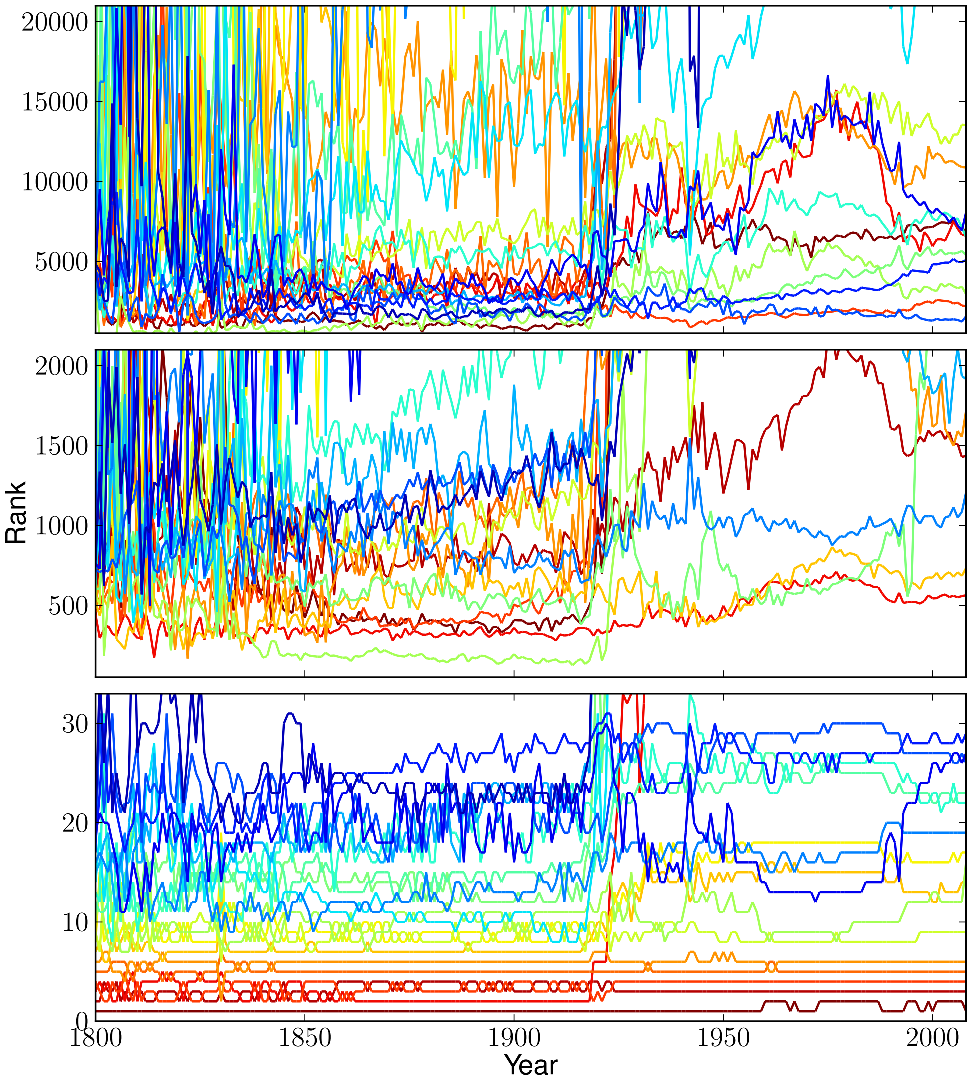

Figure S8: Rank variations in time of twenty words from three different scales for Russian.

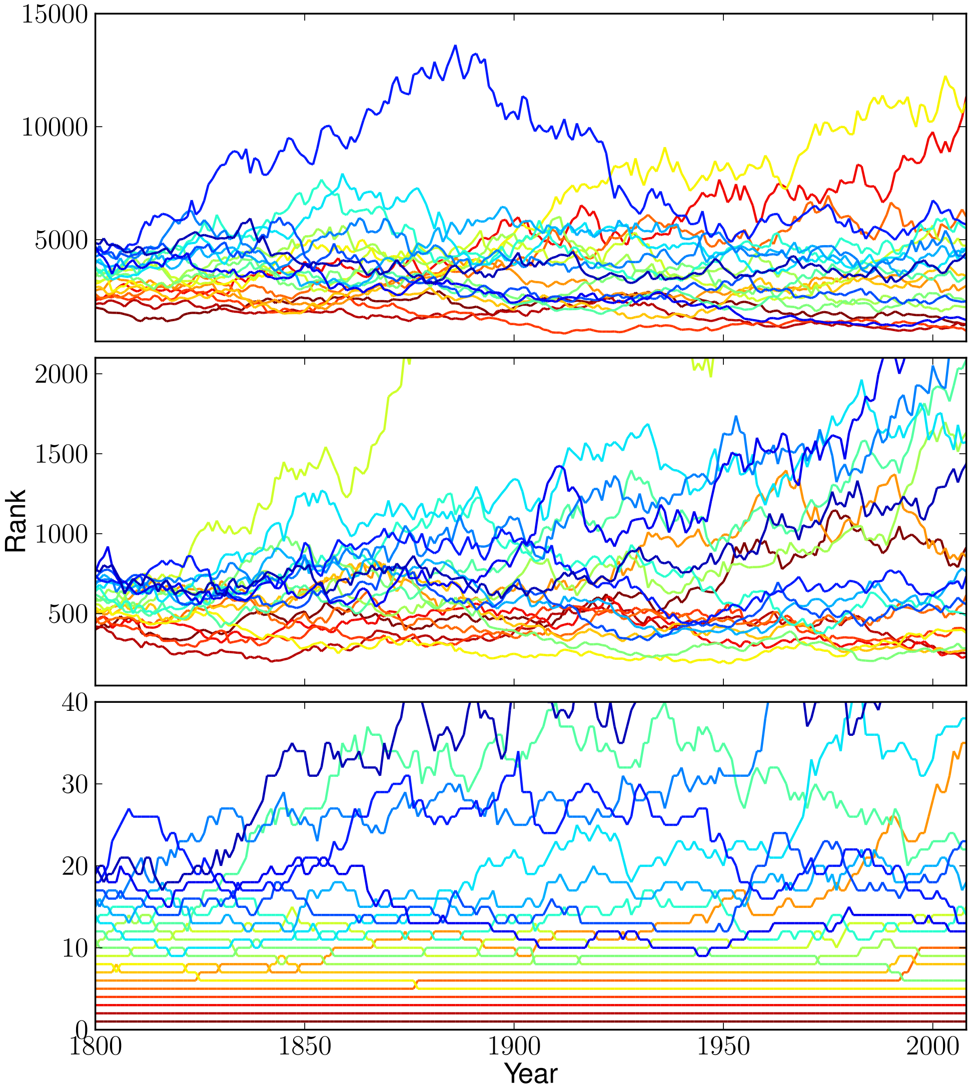

Figure S9: Rank variations in time of twenty words from three different scales for our simulated language.

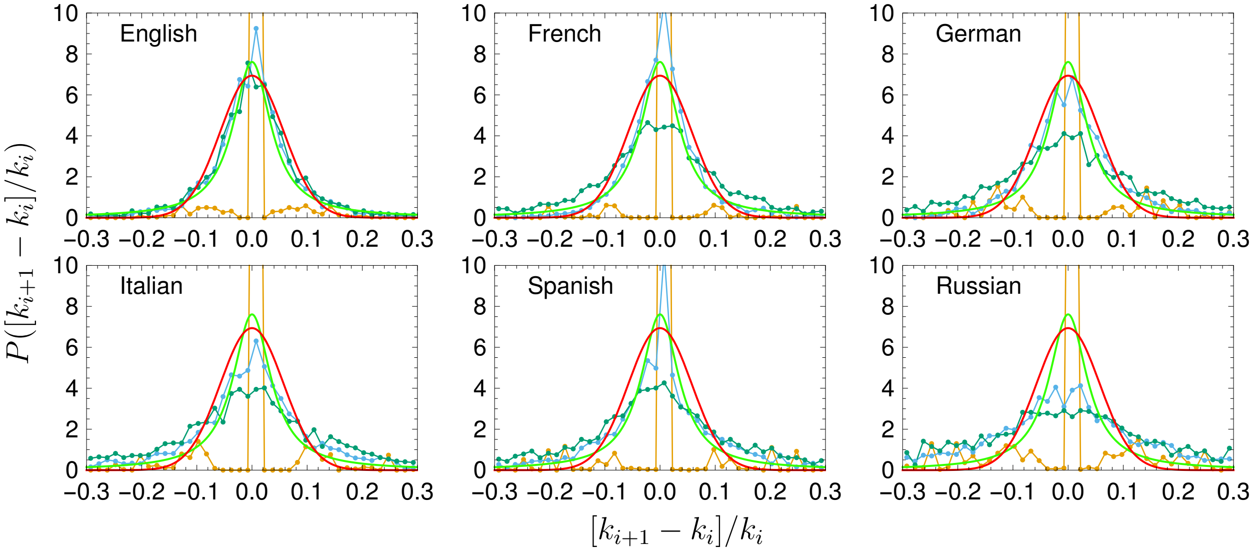

Figure S10: **Distribution of relative flights for all languages studied.** A similar plot as the one presented in figure 6 is shown for other languages. The same color coding and details are used.

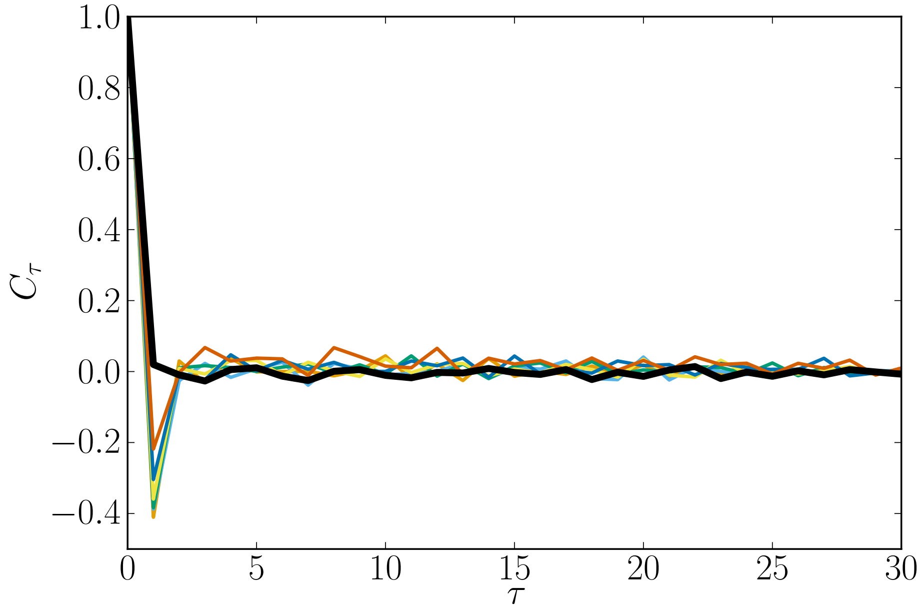

Figure S11: **Correlations for relative frequency changes** for different languages. Black line shows correlations for the simulated language.
